# Spontaneous Heterotopic Autotransplantation of Splenic Tissue: A Mimic of Pancreatic Malignancy

**DOI:** 10.14309/crj.0000000000001490

**Published:** 2024-09-11

**Authors:** Emily Fellows, Daryl Ramai, Lara Patriquin, Christopher Ko

**Affiliations:** 1 Division of Gastroenterology, Hepatology, and Nutrition, University of Utah, Salt Lake City, UT; 2 Department of Radiology, University of Utah, Salt Lake City, UT

**Keywords:** pancreatic splenosis, splenectomy, endoscopic ultrasound

## Abstract

Intra-abdominal splenosis is a rare finding which most commonly occurs following traumatic splenectomy. We present a case report of a patient who presented with abdominal pain in which peripancreatic and intrapancreatic lesions were found in the setting of mediastinal lymphadenopathy. Owing to concerns for pancreatic malignancy, we explored these lesions using endoscopic ultrasound with fine-needle biopsy (with rapid on-site evaluation). Ultimately, surgical pathologies revealed the presence of splenic tissues and the diagnosis of pancreatic splenosis.

## INTRODUCTION

Spontaneous heterotopic autotransplantation of splenic tissue, or splenosis, occurs as a result of peritoneal seeding of the splenic tissue following splenic surgeries.^1^ Splenosis commonly occurs following traumatic splenectomy and is typically found within the abdominal and pelvic cavities, with a greater proportion found within the mesentery, peritoneum, omentum, large and small bowels, and diaphragmatic surface.^1,2^

These splenic lesions are often concerning for malignancy given their imaging characteristics and anatomic locations and have prompted removal before the confirmation by pathology in some cases.^3,4^ However, because of their benign state, management through surgical resection is typically only offered in the event of symptomatic splenosis.^5^ Thus, diagnostic pause amidst workup of intra-abdominal nodules is important in preventing unnecessary medical management, as is exemplified in our case report.

## CASE REPORT

**Figure 1.**
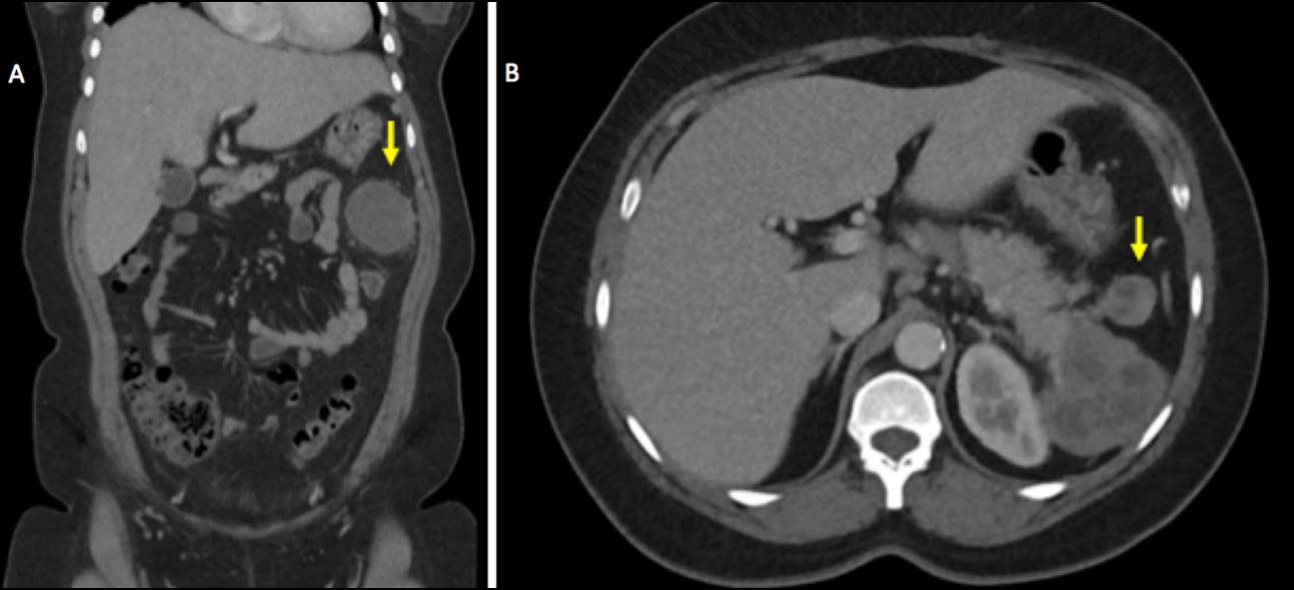
CT A/P imaging showing a heterogeneous mass (A) arising from the pancreatic tail with associated suspected lymph node (B, arrow).

A 34-year-old Spanish-speaking woman with no known medical history presented to the emergency department for the evaluation of a 3-day history of left abdominal and left flank pain with associated chills. She also reported a 2-day history of vomiting and diarrhea over an unspecified time frame. At that time, she reported a pertinent abdominal surgical history for a blood vessel in her childhood but could not recall further details. She also had a surgical history of cesarean section and hysterectomy. Her vitals were afebrile and hemodynamically stable. Laboratory results were overall noncontributory. Computed tomography of the abdomen and pelvis (CT A/P) revealed a heterogeneous, loculated mass measuring 6.3 × 6.2 × 3.8 cm, which appeared to arise from the pancreatic tail, as well as a smaller satellite mass measuring 2.3 × 2.3 cm (Figure 1). An enhancing, heterogeneous lesion measuring 5.0 × 6.7 × 6.2 cm was also seen in the left upper quadrant inferior to the pancreatic tail mass. Multiple enlarged gastrohepatic, periportal, upper retroperitoneal, and mesenteric lesions suspected to be lymph nodes were also present.

**Figure 2.**
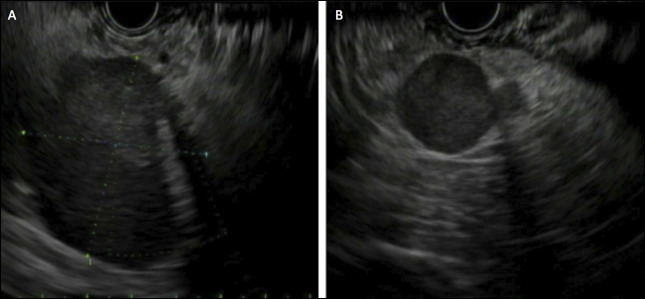
EUS imaging showing suspected pancreatic tail mass (A) and associated suspected lymph node (B).

Owing to high suspicion for malignancy, the patient underwent biopsy of the pancreatic mass by endoscopic ultrasound (EUS) (Figure 2). A 42 X 40 mm hypoechoic mass with irregular margins was identified in the pancreatic tail. The remainder of the pancreas including the upstream parenchyma and pancreatic duct was unremarkable. In addition, 2 round, hypoechoic masses (19 × 19 and 15 × 12 mm) suspected to be malignant lymph nodes and thus concerning for metastatic disease were visualized in the peripancreatic region. The endosonographic appearance was suspicious for metastatic solid pseudopapillary tumor. Fine-needle biopsies of each lesion were performed using separate 22-gauge needles. Rapid on-site evaluation was used, and preliminary cytopathologic evaluation also suggested the possibility of metastatic solid pseudopapillary tumor.

However, final pathology results of the pancreatic tail mass, peripancreatic lesions, and suspected lymph nodes were instead found to be consistent with splenic tissue suspicious for splenosis. The patient was informed of these results who then shared a pertinent surgical history of splenectomy at the age of 13 years due to splenic hypertrophy. The Radiology department was contacted afterward to confirm the absence of spleen on the initial CT A/P who revised their initial finding and confirmed asplenism. The patient's clinical picture was thus consistent with pancreatic splenosis, and she was referred to general surgery for evaluation of surgical management. By 3-month follow-up, her abdominal pain had resolved without intervention. Owing to her lack of symptoms, plans were made to monitor for recurrence of symptoms and repeat abdominal imaging in 6 months to evaluate for progression of splenosis. If the patient's symptoms recurred or imaging revealed progression, the general surgery team would discuss surgical intervention with the patient.

## DISCUSSION

Intra-abdominal splenosis is a rare finding which most commonly occurs following traumatic splenectomy.^2^ While splenosis typically is found within the pelvis, large and small bowels, peritoneum, mesentery, and omentum, isolated pancreatic splenosis has only been described in a few other case reports, with initial concern for pancreatic malignancy due to imaging characteristics.^1,4,6^ Interestingly, Li et al describe a case in which both intrahepatic splenosis and pancreatic malignancy were found amidst workup of intra-abdominal masses.^7^ Thus, splenosis can coincide with malignancy and prompts diagnostic pause amidst workup of intra-abdominal and suspicious pancreatic masses.

Our case report emphasizes the importance of diagnostic pause due to multiple findings on CT, EUS, and on-site cytology, which were concerning for metastatic solid pseudopapillary tumor. This includes both cystic and solid components visualized on CT A/P and the anatomical location of the mass at the pancreatic tail, which is seen in 61% of cases of solid pseudopapillary cancer.^8^

Ardengh et al evaluated EUS characteristics of 11 patients with pancreatic splenosis and found that pancreatic splenosis was most commonly found within the pancreatic tail and had a hypoechoic appearance, as in our patient's case.^9^ Thus, imaging alone is not sufficient in diagnostic evaluation and emphasizes the importance of final pathology in ultimately guiding medical management of pancreatic masses. Once identified, pancreatic splenosis can be managed with surgical resection in symptomatic cases.^5^ As described in the case of our patient, surgical resection was initially considered due to persistent abdominal pain, which later resolved without need for intervention.

In summary, the workup of intra-abdominal masses, specifically of pancreatic masses, is understandably broad and often anchored toward the assessment for malignancy. Pancreatic splenosis, while rare, can be a benign mimic of pancreatic malignancy and prompts thorough workup incorporating diagnostic pause as a safeguard to prevent mismanagement such as pre-emptive surgical resection before diagnostic confirmation.

## DISCLOSURES

Author contributions: E. Fellows, manuscript preparation. L. Patriquin, medical expertise. D. Ramai and C. Ko, project conceptualization and supervision. C. Ko is the article guarantor.

Financial disclosure: None to report.

Informed consent was obtained for this case report.
